# Influenza vaccine effectiveness from nine studies during drifted A(H3N2) subclade K predominance, Europe, September 2025 to January 2026

**DOI:** 10.2807/1560-7917.ES.2026.31.7.2600109

**Published:** 2026-02-19

**Authors:** Heloise Lucaccioni, Diogo FP Marques, Freja Kirsebom, Hanne-Dorthe Emborg, Mark Hamilton, Heather Whitaker, Amanda Bolt Botnen, Magda Bucholc, Francisco Pozo, Nick Andrews, Ramona Trebbien, Safraj Shahul Hameed, Karina Lauenborg Møller, Mark G O’Doherty, Jamie Lopez-Bernal, Kirsty Morrison, Simon Cottrell, Suzanne Wilton, Angela MC Rose, Esther Kissling, the European IVE group, The European IVE group, Declan T Bradley, Siobhan Murphy, Orla Crossan, Emma Dickson, Katja Hoschler, Beatrix Kele, Ross McQueenie, Kimberly Marsh, Jana Zitha, Panoraia Kalapotharakou, Simon DeLusignan, Elizabeth Button, Marie-Pierre Parsy, Mélanie Delvallee, Pierre Struyven, Arne Witdouck, Deborah De Geyter, Eveline Van Honacker, Lucie Seyler, Siel Daelemans, Nicolas Dauby, Arthur Eggerickx, Catherine Quoidbach, Martin Vandeputte, Sigi Van den Wijngaert, David Tuerlinckx, Inge Engelrelst, Marijke Reynders, Koen Magerman, Marieke Bleyen, Marlies Blommen, Natasja Detilieu, Veerle Penders, Reinout Naesens, Eva Bernaert, Bénédicte Lissoir, Catherine Sion, Sandra Koenig, Xavier Holemans, Isabel Leroux-Roels, Pascal De Waegemaeker, Silke Ternest, Anna Parys, François Dufrasne, Sarah Denayer, Juliette Thulliez, Adrien Lajot, Claire Brugerolles, Laurane De Mot, Mathil Vandromme, Peace Mpakaniye, Sébastien Fierens, Yinthe Dockx, Yves Lafort, Ralf Dürrwald, Annika Erdwiens, Kristin Tolksdorf, Ute Preuß, Irmgard Stroetmann, Barbara Biere, Marianne Wedde, Janine Reiche, Djin-Ye Oh, Virtudes Gallardo García, Inés Guiu Cañete, Ana Fernández Ibáñez, Marta Torres Juan, Eva Rivas, Luis Javier Viloria Raymundo, M.ª Angeles Rafael de la Cruz López, Beatriz Bermejo Muñoz, Jacobo Mendioroz, María del Carmen García Rodríguez, Mariano Julián Rochina, María Otero Barrós, Luis García Comas, Blanca Andreu Ivorra, Olatz Mokoroa, Miriam Blasco Alberdi, María Domínguez Padilla, Daniel Castrillejo Pérez, Ana Roldán Garrido, Álvaro Torres Lana, Lorena Díaz López, Isabel Martínez Pino, Montserrat Martinez, Ana Sofia Lameiras Azevedo, María Cecilia Puerto Hernández, Ana Carmen Ibáñez Pérez, Maria Angel Valcarcel, Jesús Vázquez Muñoz, Francisco Javier de la Vega Olías, Marcos Lozano, Gloria Pérez-Gimeno, Susana Monge, Liem Binh Luong, Odile Launay, Motolete Alaba Tanah, Louise Lefrancois, Amina Oumessoum, Célia Adjed, Yacine Saidi, Mohamed Ben Mechlia, Marina Uras, Caroline Guerrisi, Cécile Souty, Thierry Blanchon, Titouan Launay, Alessandra Falchi, Shirley Masse, Marie Chazelle, Leïla Renard, Marie-Anne Rameix-Welti, Vincent Enouf, Danielle Perez-Bercoff, Antonin Bal, Bruno Lina, Vesna Višekruna Vučina, Bernard Kaić, Sanja Kurečić Filipović, Vedrana Marić, Iva Pem Novosel, Irena Tabain, Rok Čivljak, Borna Grgić, Ivan-Krešimir Lizatović, Ivan Mlinarić, Ivana Ferenčak, Katica Čusek Adamić, Mirjana Lana Kosanović Ličina, Ivana Mihin Huskić, Diana Nonković, Morana Tomljenović, Beatrix Oroszi, Gergő Túri, Viktória Velkey, Katalin Krisztalovics, Katalin Kristóf, Krisztina Mucsányiné Juhász, Bánk Gábor Fenyves, Márta Knausz, Bernadett Burkali, István Zsolt, Zoltán Péterfi, Edit Kalamár-Birinyi, Lilla Sáfár, Júlia Tuksa, Csaba Hoffmann, Lisa Domegan, Charlene Bennett, Róisín Duffy, Margaret Fitzgerald, Karen O’Reilly, Eva Kelly, Alberto Mateo Urdiales, Antonino Bella, Simona Puzelli, Sara Piacentini, Emanuela Giombini, Marzia Facchini, Angela di Martino, Patrizio Pezzotti, Paola Stefanelli, Ligita Jancoriene, Fausta Majauskaite, Birute Zablockiene, Ausra Dziugyte, Maria Louise Borg, Iván Martínez-Baz, Aitziber Echeverría, Camino Trobajo-Sanmartín, Jesús Castilla, Ana Navascués, María Eugenia Portillo-Bordonabe, Nerea Egüés, Guillermo Ezpeleta, Itziar Casado, Manuel García Cenoz, Adam Meijer, Dirk Eggink, Mariëtte Hooiveld, Anne Huiberts, Rianne van Gageldonk-Lafeber, Raquel Guiomar, Verónica Gómez, Ausenda Machado, Camila Henriques, Nuno Verdasca, Ana Paula Rodrigues, João Almeida Santos, Licínia Gomes, Tiago Pereira Rodrigues, Daniela Dias, Rodica Popescu, Odette Popovic, Mihaela Lazăr, Dorina Ujvari, Neus Latorre-Margalef, Marlena Kaczmarek, Kate Olsson, Nathalie Nicolay, Sabrina Bacci, Anthony Nardone

**Affiliations:** 1 Epiconcept, Paris, France; 2 UK Health Security Agency, London, United Kingdom; 3 Department of Infectious Disease Epidemiology and Prevention, Statens Serum Institut, Copenhagen Denmark; 4 Public Health Scotland, Glasgow, United Kingdom; 5 Department of Virology and Microbiological Preparedness, Statens Serum Institut, Copenhagen, Denmark; 6 Public Health Agency, Belfast, United Kingdom; 7 National Centre for Microbiology, Institute of Health Carlos III, Madrid, Spain; 8 Consortium for Biomedical Research in Epidemiology and Public Health (CIBERESP), Madrid, Spain; 9 Data Integration and Analyses, Statens Serum Institut, Copenhagen, Denmark; 10 Public Health Wales, Cardiff, United Kingdom; 11 The members of the network are listed under Collaborators.; * These authors contributed equally to this work and share first authorship.; ** These authors contributed equally to this work and share last authorship.

**Keywords:** influenza, vaccine effectiveness, multicentre study, test-negative design, Europe

## Abstract

The European 2025/26 influenza season is dominated by the influenza A(H3N2) virus, with most sequenced viruses belonging to subclade K, genetically drifted from the vaccine virus, raising concerns around vaccine effectiveness (VE). Despite this, VE estimates from nine European studies (19 countries) indicate all-age influenza A VE of 25–45% for outpatient and hospital settings combined, similar to other seasons, with highest estimates among children (47–72%). Vaccination should be encouraged and complemented by other infection prevention and control measures.

The World Health Organization (WHO) recommendations for the 2025/26 northern hemisphere influenza vaccination for influenza virus type A included an A/Victoria/4897/2022 (H1N1)pdm09-like and an A/Croatia/10136RV/2023 A(H3N2)-like virus for egg-based vaccines and an A/Wisconsin/67/2022 (H1N1)pdm09-like and an A/District of Columbia/27/2023 (H3N2)-like virus for cell-based vaccines [1]. Although the influenza A(H3N2) vaccine component differs from previous seasons, the vaccine recommendations for the influenza A(H1N1)pdm09 vaccine component have remained unchanged since the 2023/24 season [2,3].

The influenza season started early in some European countries, with influenza A viruses predominating between September 2025 and January 2026. Most subtyped viruses were influenza A(H3N2), among which subclade K was dominant [4]. This subclade is highly drifted from the influenza A(H3N2) virus included in the vaccine strain (subclade J.2), suggesting potential for immune escape [5,6]. However, early influenza vaccine effectiveness (VE) estimates in November and December 2025 indicated VE against influenza A(H3N2) of ≥ 50% in individuals aged younger than 65 years [7,8].

## Design and methods of nine European influenza vaccine effectiveness studies

**Figure 1:**
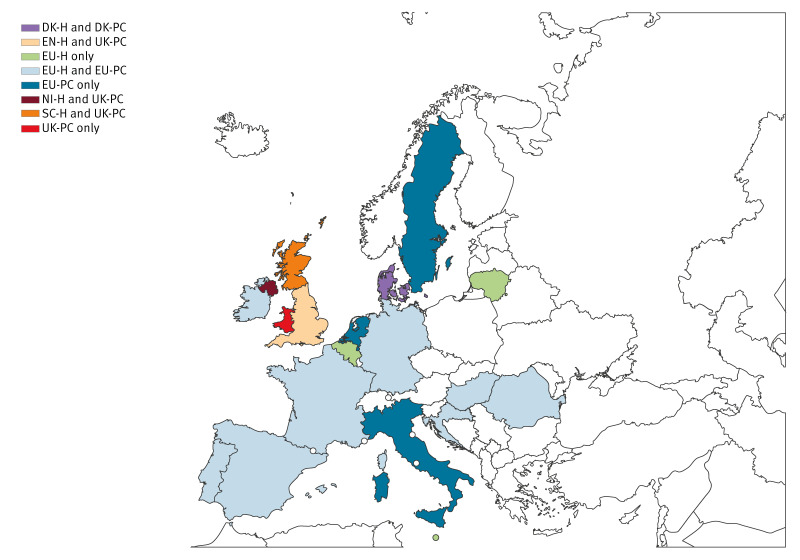
European countries contributing interim influenza vaccine effectiveness results, influenza season 2025/26 (n = 19)

We provide interim 2025/26 VE results from nine European studies, comprising six single- and three multi-country studies (19 countries in all) across outpatient and hospital settings (Figure 1). These interim estimates provide greater breadth and depth than the early 2025/26 VE estimates, to confirm early findings, guide influenza prevention and control measures for the rest of the season, and inform WHO vaccine selection recommendations for the 2026/27 season.

DK-PC/DK-H: Denmark primary care and hospital studies; EN-ED/EN-H: England emergency department and hospital studies; EU: European Union; EU-PC/EU-H: EU primary care-/hospital-based multi-country I-MOVE studies; I-MOVE: Influenza – Monitoring of Vaccine Effectiveness; NI-H: Northern Ireland hospital study; SC-H: Scotland hospital study; UK-PC: UK primary care multi-country study.

Countries contributing to EU-H but not included in the analysis (as too few cases remained after applying exclusions and restrictions): Croatia, France.

The primary care (PC) studies were conducted in Denmark (DK-PC), the United Kingdom (UK-PC) and through the I-MOVE multi-country primary care network, part of the European Union (EU) Vaccine Effectiveness, Burden and Impact Studies (VEBIS) project (EU-PC). The hospital setting (H) studies were conducted in Denmark (DK-H), England (EN-H and, for emergency departments, EN-ED), Northern Ireland (NI-H), Scotland (SC-H), and through the I-MOVE multi-country hospital network (EU-H), also part of the VEBIS project. We classified the EN-ED study as a hospital study, as 79% of patients recruited within EN-ED went on to be hospitalised. The EN-ED and EN-H studies use different administrative datasets, but there is some (50–73%) overlap among patients.

All studies used the test-negative design, using methods as described previously [9-13]. Key methods are outlined in Tables 1 and 2.

**Table 1:** Summary of methods for the European interim influenza vaccine effectiveness studies in the primary care and emergency settings, influenza season 2025/26 (n  = 16 countries)

Study characteristics	DK-PC	EU-PC	UK-PC
Period	13 Oct 2025–16 Jan 2026 ISO weeks 42–03	29 Sep 2025–13 Jan 2026 ISO weeks 40–03	29 Sep 2025–4 Jan 2026 ISO weeks 40–01
Setting	Non-hospitalised patients^a^	Primary care	Primary care
Location	DK	DE, ES, FR, HR, HU, IE, IT, NA, NL, PT, RO, SE	EN, NI, SC, WA
Design	TND	TND	TND
Data source(s)	Data linkage of Danish Microbiology Database, the Danish Vaccination Register and the Danish National Discharge Register	Sentinel physicians and laboratories, linkage to vaccine registries	Sentinel physicians and laboratories; in some sites data linkage to vaccine registries
Age groups of study population	All ages	≥ 6 months	≥ 2 years
Case definition for patient recruitment	Sudden onset of symptoms with fever, myalgia and respiratory symptoms^b^	EU ARI^c^ or EU ILI^d^	ARI
Selection of patients	At practitioner's/clinician's judgement	Systematic^e^	At practitioner's/clinician's judgement
Vaccine types used among controls^f^	Ages 0–17 years (n = 52): 100% IIVe; ages 18–64 years (n = 1,587): 99.5% IIVe, 0.5% aIIV; ages ≥ 65 years (n = 3,573): 80% aIIV, 20% IIVe	Ages 0–17 years (n = 582): 51% IIVe, 36% LAIV, 1% other, 1% IIVc, 12% unknown; ages 2–17 years (n = 365): 58% LAIV, 27% IIVe, 1% other, 14% unknown; ages 18–64 years (n = 490): 59% IIVe, 11% IIVc, 10% aIIV, 2% other, 2% IIV-HD, 16% unknown; ages ≥ 65 years (n = 691): 39% aIIV, 25% IIVe, 12% IIV-HD, 3% IIVc, 3% other, 17% unknown; target group (n = 1,553): 41% IIVe, 20% aIIV, 12% LAIV, 6% IIV-HD, 4% IIVc, 2% other, 14% unknown	Ages 2–17 years (n = 662): 92% LAIV, 7% IIVc, unknown 1%; ages 18–64 years (n = 1,082): < 1% IIVe, 79% IIVc, 6% IIVr, 5% aIIV, < 1% IIV-HD, 9% unknown; ages ≥ 65 years (n = 1,309): < 1% IIVe, 2% IIVc, 6% IIVr, 86% aIIV, < 1% IIV-HD, 6% unknown
Variables of adjustment	Age group, sex, presence of chronic conditions, calendar time as month (Oct–Jan) or if possible, week	Age (modelled as RCS, age group or linear term depending on analysis), sex, presence of chronic conditions, time (onset date as RCS or ISO week) and study site	Age group, sex, country, clinical risk status, calendar time as week (spline)

aIIV: adjuvanted IIV; ARI: acute respiratory infection; DE: Germany; DK-PC: Denmark primary care studies; EN: England; ES: Spain; EU: European Union; EU-PC: EU primary care multicentre I-MOVE studies; FR: France; HR: Croatia; HU: Hungary; IE: Ireland; IIV: inactivated influenza vaccine; IIVc; cell-based IIV; IIVe: egg-based, standard dose, non-adjuvanted IIV; IIV-HD: high-dose IIV; IIVr: recombinant IIV; ILI: influenza-like illness; I-MOVE: Influenza – Monitoring of Vaccine Effectiveness in Europe; LAIV: live attenuated influenza vaccine; ISO: International Organization for Standardization; NA: Navarre region, Spain; NI: Northern Ireland; PT: Portugal; RCS: restricted cubic spline; RO: Romania; SC: Scotland; SE: Sweden; TND: test-negative design; UK-PC: UK multicentre primary care study; WA: Wales.

^a^ Patients are seen by the general practitioner, but also in emergency care.

^b^ This is the case definition for patient recruitment by sentinel general practitioners within DK-PC who follow the EU-ILI case definition (sudden onset of symptoms, AND at least one of: fever > 38°C, feverishness, malaise, headache, myalgia, AND at least one of: cough, sore throat, shortness of breath).

^c^ The EU-ARI definition is sudden onset of symptoms AND ≥ 1 of cough, sore throat, shortness of breath or coryza AND a clinician’s judgement that the illness is due to an infection.

^d^ The EU-ILI definition is sudden onset of symptoms AND ≥ 1 of: fever or feverishness, malaise, headache, myalgia AND ≥ 1 of cough, sore throat, shortness of breath. Most EU-PC sites recruit according to the EU-ARI case definition, although there are some site-specific differences in ARI (and/or ILI) definitions for recruitment.

^e^ In EU-PC, the approach used to select participants to include in the study varied across sites, e.g. selection of all patients meeting the case definition; systematic selection overall (e.g. the first five patients of the week meeting the case definition); systematic selection by age group (e.g. the first two patients of the week meeting the case definition aged < 65 years and the first two patients aged ≥ 65 years).

^f^ Vaccine viruses are egg-based, non-adjuvanted and administered intramuscularly unless otherwise specified.

**Table 2:** Summary of methods for the European interim influenza vaccine effectiveness studies in the hospital setting, influenza season 2025/26 (n  = 13 countries)

Study characteristics	DK-H	EN-ED	EN-H	EU-H	SC-H	NI-H
Period	13 Oct 2025–16 Jan 2026 ISO weeks 42–03	29 Sep 2025–4 Jan 2026 ISO weeks 40–01	29 Sep 2025–14 Dec 2025 ISO weeks 40–50	16 Sep 2025–11 Jan 2026 ISO weeks 38–02	28 Sep 2025–21 Jan 2026 ISO weeks 39–04	29 Sep 2025– 10 Jan 2026 ISO weeks 40–02
Setting	Hospital	Emergency care attendance	Hospital	Hospital	Hospital	Emergency hospital admissions
Location	DK	EN	EN	97 hospitals in BE, DE, ES, HU, IE, LT, MT, NA, PT, RO	SC	NI
Design	TND	TND	TND	TND	TND	TND
Data source(s)	Data linkage of Danish Microbiology Database, the Danish Vaccination Register and the Danish National Discharge Register	Data linkage of laboratory surveillance, the Immunisations Information System IIS, and national emergency care attendance data ECDS	Data linkage of laboratory surveillance, the Immunisations Information System IIS, and the Secondary Uses Service SUS	Hospital charts, vaccine registers, interviews with patients, laboratory records	National patient-level dataset based on GP records, Electronic Communication of Surveillance in Scotland ECOSS (all virology testing national database), Rapid Preliminary Inpatient Data RAPID (Scottish hospital admissions data), National Records of Scotland NRS (death certification), National Clinical Data Store NCDS (vaccination events in Scotland)	Linkage of vaccination status from the Northern Ireland Vaccine Management System, influenza tests from the NI regional surveillance system, and administrative admissions data from Health and Social Care information system Epic
Age groups of study population	All ages	≥ 2 years	≥ 2 years	≥ 6 months	All ages	Adults ≥ 18 years
Case definition for patient recruitment	Sudden onset of symptoms with fever, myalgia and respiratory symptoms	Influenza test up to 14 days before or within 2 days after an emergency department attendance (non-injury related)	Influenza test up to 14 days before or within 2 days after an ARI ^a^-coded hospital visit	Severe ARI (hospitalised person with fever cough, or shortness of breath) at admission or within 48 h after admission); some countries recruit those with fever or cough; some include only those with fever and cough	Influenza test 14 days before admission or within 48 h of admission; limited to emergency hospitalisation (unplanned admission)	Influenza test up to 14 days before or within 2 days after an emergency department admission
Selection of patients	At practitioner's/ clinician's judgement	Exhaustive (all patients who fit the case definition above and are captured via the linkage of the named datasets)	Exhaustive (all patients who fit the case definition above and are captured via the linkage of the named datasets)	Exhaustive (BE, DE, HU, IE, LT, MT, NA, PT, RO) and systematic (ES: exhaustive on either 1 or 2 days per week, depending on workload)	Exhaustive (all patients who fit the case definition above and are captured via the linkage of the named datasets)	Exhaustive (all patients who fit the case definition and are captured via the linkage of the named datasets)
Vaccine types used among controls^b^	Ages 0–17 years (n = 15): 100% IIVe; ages 18–64 years (n = 695): 99% IIVe, 1% aIIV; ages ≥ 65 years (n = 6,660): 89% aIIV, 11% IIVe	Ages 2–17 years (n = 4,045): 84% LAIV, 12% IIVc, 4% unknown; ages 18–64 years (n = 7,817): 72% IIVc, 11% IIVr, 2% IIVe, 9% aIIV, 5% unknown; ages ≥ 65 years (n = 29,858): 4% IIVc, 2% IIV-HD, 79% aIIV, 10% IIVr, 5% unknown	Ages 2–17 years (n = 2,697): 79% LAIV, 16% IIVc, 5% unknown; ages 18–64 years (n = 1,195): 74% IIVc, 11% IIVr, 1% IIVe, 9% aIIV, 5% unknown; ages ≥ 65 years (n = 4,964): 4% IIVc, 2% IIV-HD, 79% aIIV, 10% IIVr, 5% unknown	Ages 6 months–17 years (n = 240): 61% IIVe, 29% LAIV, 1% IIVc, 9% unknown; ages 18–64 years (n = 175): 62% IIVe, 13% aIIV, 7% IIVc, 4% IIV-HD, 14% unknown; ages ≥ 65 years (n = 1,392): 46% IIVe, 27% aIIV, 15% IIV-HD, 2% IIVc, 10% unknown	Ages 2–4 years (n = 199): 14.6% IIVc, 85.4% LAIV; ages 5–17 years (n = 290): 12.8% IIVc, 87.2% LAIV; 18–64 years (n = 988): 4.9% aIIV, 95.0% IIVc, 0.1% LAIV; ages ≥65 years (n = 6,299): 99.6% aIIV, 0.4% IIVc	Ages 18–64 years (n = 84): 34.2% IIVc, 0.1% IIVe, 1% aIIV, 0.6 LAIV, 64.1% unknown; ages ≥ 65 years (n = 434): 2.9% IIVc, 0.02% IIVe, 42.6% aIIV, 54.5% unknown
Variables of adjustment	Age group, sex, presence of chronic conditions, calendar time as month (Oct–Jan) or if possible, week	Age group, region, clinical risk status, calendar time as week (spline)	Age group, region, clinical risk status, calendar time as week (spline)	Age (modelled as RCS, age group or linear term depending on analysis), sex, presence of chronic conditions, time (onset date as RCS or month of onset as categorical term) and study site	Age (spline), sex, number of clinical risk groups (0, 1, 2, 3, 4, ≥ 5), time (days, spline), setting (sample obtained in community or hospital), vaccine eligibility status, immunosuppression status, deprivation quintile (SIMD)	Age (spline), sex, week (spline), and Health and Social Care Trust

aIIV: adjuvanted IIV; ARI: acute respiratory infection; BE: Belgium; DE: Germany; DK-H: Denmark hospital studies; EN: England; EN-ED: England emergency department study; EN-H: England hospital study; ES: Spain; EU: European Union; EU-H: EU hospital multicentre I-MOVE studies; GP: general practitioner; H: hospital; HU: Hungary; IE: Ireland; IIV: inactivated influenza vaccine; IIVc; cell-based IIV; IIVe: egg-based, standard dose, non-adjuvanted IIV; IIV-HD: high-dose IIV; IIVr: recombinant IIV; ILI: influenza-like illness; I-MOVE: Influenza – Monitoring of Vaccine Effectiveness in Europe; LAIV: live attenuated influenza vaccine; ISO: International Organization for Standardization; LT: Lithuania; MT: Malta; NA: Navarre region, Spain; NI: Northern Ireland; NI-H: Northern Ireland hospital study; PT: Portugal; RCS: restricted cubic spline; RO: Romania; SC: Scotland; SC-H: Scottish hospital study; TND: test-negative design.

^a^The EU-ARI definition is sudden onset of symptoms AND ≥ 1 of cough, sore throat, shortness of breath or coryza AND a clinician’s judgement that the illness is due to an infection.

^b^ Vaccine viruses are egg-based, non-adjuvanted and administered intramuscularly unless otherwise specified.

Patients were recruited prospectively in three studies (EU-H, EU-PC and UK-PC) and through electronic database linkage in six studies (DK-H, DK-PC, EN-ED, EN-H, NI-H, SC-H).

In the DK-H, DK-PC, EU-PC and UK-PC settings, patients presenting with influenza-like illness (ILI) or acute respiratory infection (ARI) symptoms provided specimens. In EU-H, hospitalised patients with severe acute respiratory infection (SARI) were swabbed. For EN-ED, EN-H, NI-H, and SC-H, all patients tested for influenza virus were presumed to have at least one ARI symptom. Swabbing strategies varied across studies and included testing all eligible patients, systematic sampling, or physician discretion (Tables 1 and 2).

Influenza virus infection was confirmed using RT-PCR assays targeting influenza A and B viruses, followed by subtyping for influenza A viruses. Cases were defined as individuals RT-PCR-positive for any influenza virus, while controls tested negative for all influenza viruses. The age of recruited participants varied between studies (Tables 1 and 2). For genetic characterisation, several countries (10 in EU-PC, five in EU-H, two in UK-PC, and Denmark) selected either all or a random subset of influenza virus-positive specimens for haemagglutinin gene segment and/or whole genome sequencing. Phylogenetic analyses assigned viruses to clades and subclades; sequencing data from DK-PC and DK-H were combined for the genetic description.

Vaccination status was classified based on receipt of the 2025/26 seasonal influenza vaccine at least 14 days before symptom onset; those vaccinated 1–13 days before symptom onset or with unknown vaccination dates were excluded.

## Virological characterisation of influenza viruses within the studies

**Figure 2:**
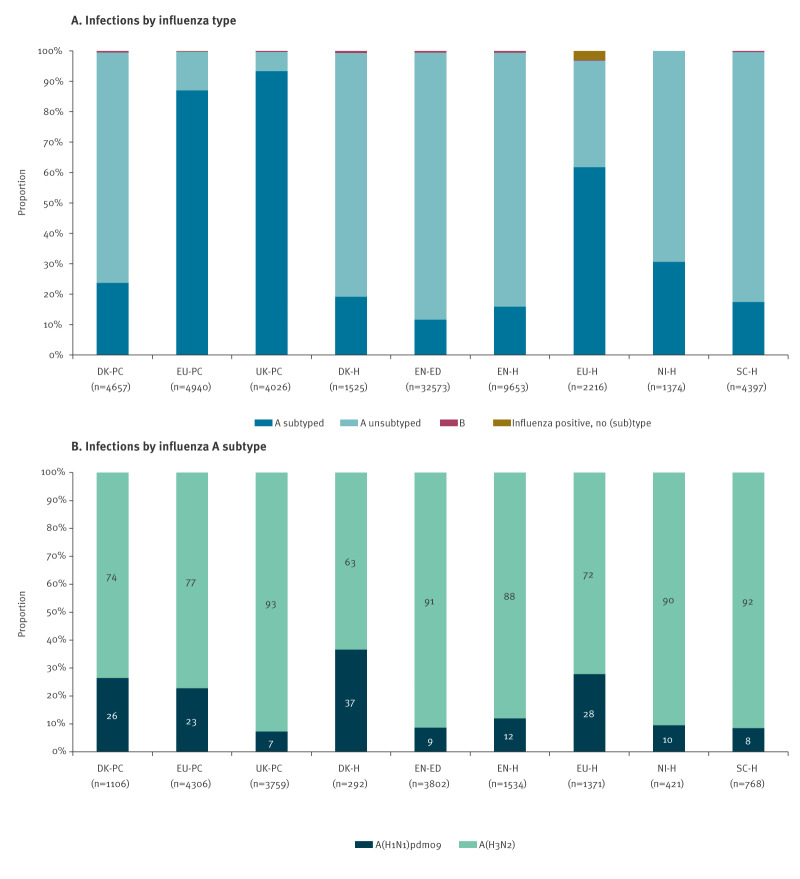
Proportion of influenza virus type and subtype infections, nine European studies, interim influenza season 2025/26 (n = 65,361)^a^

DK-H: Denmark hospital study; DK-PC: Denmark primary care study; EN-ED: England emergency department study; EN-H: England hospital study; EU: European Union; EU-H: EU hospital multicentre I-MOVE study; EU-PC: EU primary care multicentre I-MOVE study; I-MOVE: Influenza – Monitoring of Vaccine Effectiveness in Europe; NI-H: Northern Ireland hospital study; SC-H: Scottish hospital study; UK-PC: UK multicentre primary care study.

^a^ Includes 49 influenza A and B co-infections in EN-ED, 13 in EN-H, three in EU-H and two in EU-PC; eight A(H1N1)pdm09 and A(H3N2) co-infections in EN-ED, two in EN-H, three in EU-H and six in EU-PC. In addition, for EU-H, 10 cases from four study sites with < 10 cases were excluded from the A(H1N1)pdm09 analyses; for EU-PC, eight cases from two study sites with < 10 cases were excluded for the A(H1N1)pdm09 analyses.

Within all participating studies, most infections were due to influenza A virus, with < 1% (312/65,361) influenza B virus infections and 67 infections of unknown influenza virus type (Figure 2A). Among influenza A infections where subtype was known (27%; 17,359/64,982), 85% were influenza A(H3N2) viruses, with study-specific proportions ranging from 63% to 93% (Figure 2B).

Sequencing results were available from five studies. Among genetically characterised influenza A(H3N2) viruses, 94% (1,319/1,398) belonged to subclade K, with proportions ranging from 84% to 97% across contributing sites (Table 3).

**Table 3:** Genetic characterisation of influenza viruses by (sub)clade, five European studies, interim influenza season 2025/26 (n = 1,730)^a^

Influenza A viruses	Clade	Subclade	DK-H/ DK-PC^b^		EU-H		EU-PC		UK-PC	
			n	%	n	%	n	%	n	%
Influenza A(H3N2)										
N included in analysis			998	100	986	100	3,317	100	3,486	100
N genetically characterised			107	11	133	13	337	10	821	24
Genetic characterisation results										
A/Thailand/8/2022-like	2a.3a.1	J	0	0	0	0	0	0	6	1
A/Croatia/10136RV/2023-like	2a.3a.1	J.2	5	5	3	2	4	1	0	0
A/Lisboa/216/2023-like	2a.3a.1	J.2.2	1	1	2	2	3	1	1	0
A/Netherlands/10685/2024-like	2a.3a.1	J.2.3	2	2	3	2	9	3	4	0
A/Singapore/GP20238/2024-like	2a.3a.1	J.2.4	9	8	4	3	8	2	13	2
A/Norway/8765/2025-like	2a.3a.1	K	90	84	121	91	313	93	795	97
A/Victoria/211/2025-like	2a.3a.1	J.2.5	0	0	0	0	0	0	2	0
Influenza A(H1N1)pdm09										
N included in analysis			400	100	369	100	967	100	273	100
N genetically characterised			87	22	90	24	136	14	52	19
Genetic characterisation results										
A/Hungary/286/2024-like	5a.2a	C.1.9.3	0	0	1	1	0	0	1	NC
A/Missouri/11/2025-like	5a.2a.1	D.3.1^c^	87	100	89	99	136	100	51	NC
	5a.2a.1	D.3.1.1	33	38	81	90	130	96	NK	NC

DK-H: Denmark hospital study; DK-PC: Denmark primary care study; EU: European Union; EU-H: EU hospital multicentre I-MOVE study; EU-PC: EU primary care multicentre I-MOVE study; I-MOVE: Influenza – Monitoring Vaccine Effectiveness in Europe; NC: not calculated (percentages not shown where denominators < 60); NK: not known; UK-PC: UK multicentre primary care study.

^a^ Genetic characterisation results not available from the England, Northern Ireland and Scotland hospital studies, nor from the England emergency department study.

^b^ DK-H and DK-PC samples are combined.

^c^ Viruses belonging to D.3.1 and subclades (including D.3.1.1).

All but two genetically characterised influenza A(H1N1)pdm09 viruses belonged to clade D.3.1 or its subclades (Table 3), with subclade D.3.1.1 representing 78% (244/312) of D.3.1 viruses for which the subclade was known across study sites. Subclade D.3.1.1 is characterised by the R113K, A139D, E283K (and most often K302E) substitutions.

## Study population characteristics

To facilitate interpretation of VE estimates, characteristics of the study populations are provided in Supplementary Tables S1–S9. Among primary care studies, the median age of controls ranged from 34 to 50 years, whereas in hospital-based studies it ranged from 67 to 75 years. Among influenza A(H3N2) cases, the median age ranged from 19 to 46 years in primary care studies and from 19 to 73 years in hospital studies. Median ages for influenza A(H1N1)pdm09 cases ranged from 29 to 48 years and from 53 to 75 years in primary care and hospital studies, respectively.

In hospital-based studies, the majority of participants had at least one chronic condition, with proportions ranging from 65% to 86% among controls and from 45% to 74% among all cases. In primary care studies, the proportion of controls with at least one chronic condition ranged from 25% to 40%, compared with 16% to 30% among all cases.

Target groups for influenza vaccination varied across countries included in the analysis. These groups comprised individuals with underlying medical conditions, older adults above a country-specific age threshold (60 or 65 years) and, in many countries, pregnant people, care home residents, health and social care workers, carers and close contacts of immunosuppressed individuals. In the UK, children from 2 to 15, 16 or 17 years (depending on school year and nation) were part of the target group for vaccination. In EU-H and EU-PC, only some studies included children in the vaccination target group regardless of medical conditions, with targeted age ranges varying between countries. In one EU-H country, a universal vaccination recommendation was in place.

## Vaccine effectiveness

With limited influenza B virus circulation, influenza A VE reflects the overall effectiveness of influenza vaccination in the population; we provide estimates against any influenza in Supplementary Figure S1.

**Figure 3:**
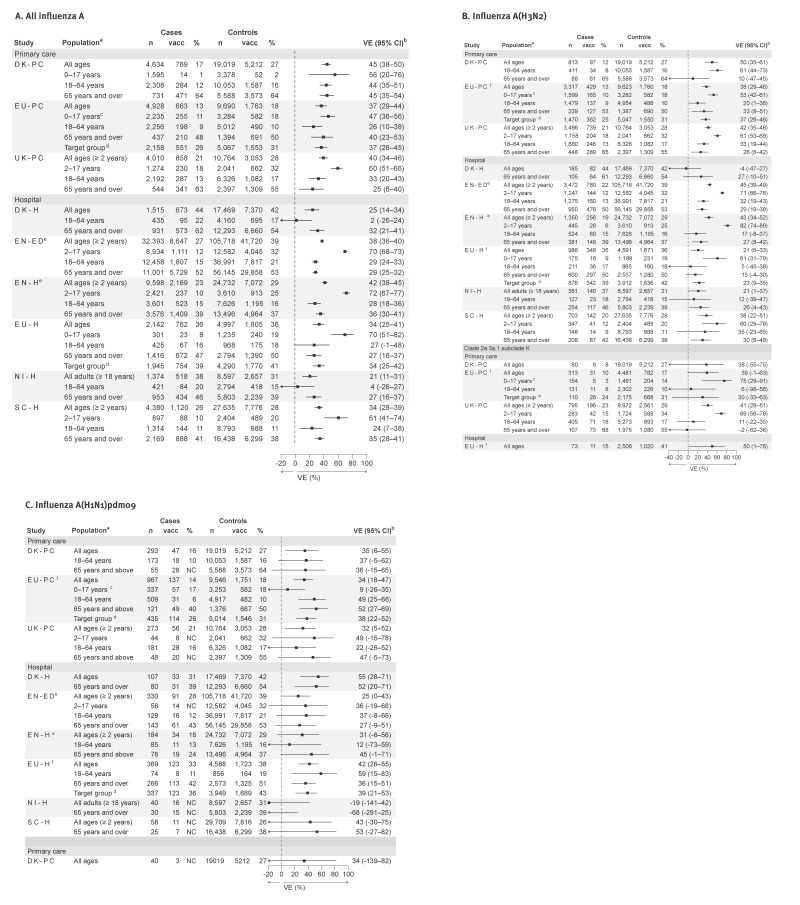
Interim vaccine effectiveness estimates against influenza A, A(H3N2)^†^ and A(H1N1)pdm09, by age group and target population, nine European studies, interim influenza season 2025/26 (n = 275,785)

CI: confidence interval; DK-H/PC: Denmark hospital/primary care studies; EN-ED: England emergency department study; EN-H: England hospital study; EU: European Union; EU-H/PC: EU hospital/primary care multicentre I-MOVE studies; H: hospital; I-MOVE: Influenza – Monitoring Vaccine Effectiveness in Europe; PC: primary care; NI-H: Northern Ireland hospital study; SC-H: Scottish hospital study; UK-PC: UK multicentre primary care study; VE: vaccine effectiveness.

^a^ Age- or target group-specific VE estimates were not available for some study sites owing to insufficient sample size.

^b^ Adjustment variables are described in Tables 1 and 2.

^c^ In EU-PC, the age group 0–17 years includes children aged ≥ 6 months to 17 years.

^d^ Target groups for seasonal influenza vaccination were defined locally by each study site.

^e^ There is a 50–73% overlap among EN-ED and EN-H patients.

^f^ For EU-PC: two study sites with < 10 A(H1N1)pdm09 cases were excluded from the A(H1N1)pdm09 VE analysis (eight cases). For EU-H: four study sites were excluded from the A(H1N1)pdm09 analyses (10 cases).

Interim influenza A VE among studies providing estimates for all ages (both settings) ranged from 25% to 45% (Figure 3), with higher estimates among children < 18 years (47–72%). Among adults aged 18–64 years, VE ranged from 2% (DK-H) to 29% (EN-ED) in the hospital setting and from 26% (EU-PC) to 44% (DK-PC) in the primary care setting. VE among older adults (≥ 65 years) ranged from 25% to 45%.

### Influenza A(H3N2)

The proportion of influenza A(H3N2) among all subtyped viruses ranged 63–93% across study sites. All-age VE against influenza A(H3N2) ranged from 38% to 50% across six study sites, with lower estimates of around 21% in EU-H (Figure 3). No effectiveness against influenza A(H3N2) was observed in DK-H; however, the numbers were small. Vaccine effectiveness among children was consistently high (51–82%), consistently higher than estimates among adults aged ≥ 18 years. Among seven of eight studies providing influenza A(H3N2) estimates for adults aged 18–64 years, VE ranged from 5% to 35%, with a higher estimate of 61% in DK-PC. Among adults aged ≥ 65 years, VE against influenza A(H3N2) ranged from 26% to 33% in seven studies, with lower estimates in two (10% and 15%). Estimates among younger and older adults were generally similar, differing between 3% and 14% in seven of the eight studies where estimates were available.

All-age estimates against subclade K were 38% and 41%^†^ in the three primary care studies, and 50% in EU-H (Figure 3).

### Influenza A(H1N1)pdm09

The VE against influenza A(H1N1)pdm09 among all ages ranged from 25% to 35% in most study sites, with higher effectiveness observed in SC-H (43%) and DK-H (55%) (Figure 3). The influenza A(H1N1)pdm09 VE ranged from 9% to 49% in children, but only three studies had enough sample size to provide estimates against this subtype for those < 18 years. Similarly, among younger adults (18–64 years) across both settings, sample size was only enough to provide estimates from five of the nine studies, with VE point estimates from 12% to 59%.

Among older adults (≥ 65 years), influenza A(H1N1)pdm09 VE point estimates ranged from 45% to 53% in five studies, but was lower in EN-ED (27%), EU-H (36%), DK-PC (36%); in NI-H, no effectiveness was observed, with a very small number of 30 cases.

DK-PC reported an all-age VE against clade 5a.2a.1, subclade D.3.1, of 34% (95% CI: −139 to 82).

### Influenza B

Influenza B circulation was minimal. The VE against influenza B, appended in Supplementary Figure S2, ranged from −10% to 62% across age groups and settings; however, estimates need to be interpreted with caution due to small numbers.

## Discussion

In a season dominated by influenza A(H3N2), results from nine European studies indicate overall low–moderate VE against influenza A and its subtypes, with vaccination preventing from approximately one-third to nearly one-half of influenza-associated outcomes among vaccinated individuals. The VE was generally higher in children than in adults. 

Three studies (EN-ED, EU-PC and SC-H) published early-season 2025/26 influenza A VE estimates [5-7], allowing comparison with these interim VE estimates and showing differences of ≤ 7 percentage points, except in SC-H for 2–17-year-olds (17 percentage point difference). For NI-H, interim results among older adults were 7 percentage points lower than early published results (pooled with Scotland and Wales) [14]. While all of these differences were well within sampling uncertainty, the estimates presented in this manuscript are all consistently lower. 

Differences in influenza A subtype-specific VE and variations in subtype distribution limit direct comparability between studies. Against influenza A(H3N2), results for VE among children aligned with early-season estimates [7,8,14], while early VE estimates for adults aged 18–64 years in EU-PC and EN-ED were 28–37% higher than in this interim analysis [7,8]. More research is needed to better understand why VE was low in adults aged 18–64 years, including the potential role of birth cohort-specific effects [15]. Many countries only recommend vaccination in specific groups in this age range (e.g. those with co-morbidities, healthcare workers); the low VE could partly be due to vaccinated and unvaccinated populations being from different populations. Sample sizes for subtype-specific VE estimates were limited in some studies. As influenza A(H3N2) predominated during the study period, the overall influenza A VE may largely reflect VE against A(H3N2). The results for VE against subclade K are comparable with 2025/26 interim results from primary care settings in Canada at 37% [15].

The influenza A(H3N2) VE findings are in line with, or slightly higher than, VE against A(H3N2) reported in previous seasons [16,17]. This reinforces the message from early season VE estimates, that despite substantial antigenic changes in circulating A(H3N2) viruses, the vaccine provides meaningful protection this season. 

Our VE estimates are generally lower than those typically reported for VE against A(H1N1)pdm09 [11,16,18,19]. An early influenza A(H1N1)pdm09 VE estimate in EU-PC was 16%, with wide confidence intervals [7]; 18% lower than the EU-PC estimate in this interim analysis. Although the vaccine was clade-matched to circulating viruses, the single clade 5a.2a.1, subclade D.3.1 VE was low (34%). Reduced VE estimates against clade 5a.2a.1 with the same vaccine were reported in 2023/24 and 2024/25 [11,20,21], although clade 5a.2a.1 viruses have evolved over time and VE may be difficult to compare across seasons. However, as A(H1N1)pdm09 accounted for a minority of circulating viruses during the study period, limited sample size resulted in low precision around the estimates; end-of-season analyses with larger sample size may allow more robust conclusions.

While ferret antisera raised against the cell-based 2025/26 northern hemisphere influenza A(H1N1)pdm09 vaccine showed good recognition of circulating viruses, human serology studies have shown substantial reduction in post-vaccination geometric mean titres [22]. For influenza A(H3N2), post-infection ferret antisera raised against vaccine viruses recognised circulating influenza A(H3N2) viruses poorly [22], however a human serology study has reported more limited reductions [23], consistent with our VE against influenza A(H3N2). This highlights the need to further evaluate the relevance of antigenic characterisation assays based on ferret antisera as a proxy for vaccine performance assessment in the context of complex human immunological landscapes.

Across studies, VE point estimates in children, where available, were lower in DK-PC, EU-PC and EU-H compared with UK estimates (EN-ED, EN-H, SC-H, UK-PC). While this may be random variation, it could also reflect a greater use of live attenuated influenza vaccine (LAIV) in the UK. The considerable use of enhanced vaccine among adults across all studies may have contributed to sustaining VE in the context of highly drifted circulating viruses compared with the vaccine strain.

A particular challenge of this manuscript is that we summarise studies across Europe that have some methodological differences, and heterogeneous study populations. While all studies presented used the TND, there are differences in patient recruitment and case definitions used. Different types of vaccines were used in different proportions across studies, and we also observe some geographical differences in circulating genetic variants. Other differences between studies and study populations include differences in season start dates and vaccination campaign timings, leading to different times since vaccination among participants, as well as different immune landscapes and vaccination histories between the populations in each country. However, a compilation of European estimates is useful to understand variation in VE across Europe. Another limitation is that sample size was small for some sub-analyses, resulting in low precision around the point estimates. As with all observational studies, unmeasured confounding cannot be ruled out. In six studies, only a small proportion of viruses were subtyped (12–31%), and selection of samples to be subtyped could have introduced bias. However, lack of subtyping was largely driven by differences in laboratory practice (e.g. many UK hospital laboratories did not routinely perform subtyping) rather than patient characteristics and therefore, substantial selection bias is considered unlikely.

## Conclusions

Our interim findings will contribute to the evidence base to inform the WHO vaccine composition meeting for the northern hemisphere influenza vaccine strain selection 2026/27, scheduled for 23–26 February 2026. Although VE was moderate, it was higher than may have been anticipated given genetic and associated antigenic characteristics of circulating viruses. As influenza activity is ongoing, our results validate previous recommendations and support continued efforts to deliver influenza vaccination in eligible groups, as well as strengthening infection prevention and control measures.

## Data Availability

Data are available from the corresponding author on request. The 1,763 sequences generated in connection with this analysis have been submitted to GISAID.

## ^†^Authors’ correction

In the original publication, the authors had inadvertently omitted subclade K estimates from the UK-PC study. These were included after publication by adding a UK-PC section in Figure 3 panel B under Clade 2a.3a.1 subclade K, Primary care. The corresponding percentage was updated from 39% to 41% in the main article text: “All-age estimates against subclade K were 38% and 41% in the three primary care studies, and 50% in EU-H (Figure 3).” These corrections were made on 16 March 2026 at the request of the authors.

## Supplementary Data

499000 Supplement
