# Author’s correction for Euro Surveill. 2026;31(7)

**DOI:** 10.2807/1560-7917.ES.2026.31.11.260319c

**Published:** 2026-03-19

**Authors:** Eurosurveillance editorial team

**Affiliations:** 1 European Centre for Disease Prevention and Control (ECDC), Stockholm, Sweden

In the article ‘Influenza vaccine effectiveness from nine studies during drifted A(H3N2) subclade K predominance, Europe, September 2025 to January 2026‘ by Lucaccioni et al., published on 19 February 2026, the authors inadvertently omitted subclade K estimates from the UK-PC study. These were included after publication by adding a UK-PC section in Figure 3 panel B under Clade 2a.3a.1 subclade K, Primary care. The corresponding percentage was updated from 39% to 41% in the main article text: “All-age estimates against subclade K were 38% and 41% in the three primary care studies, and 50% in EU-H (Figure 3).” These corrections were made on 16 March 2026 at the request of the authors.

