# Supplementary material for: Influenza vaccine effectiveness from nine studies during drifted A(H3N2) subclade K predominance, Europe, September 2025 to January 2026
Source: Euro Surveill. 2026 Feb 19;31(7):2600109. doi: 10.2807/1560-7917.ES.2026.31.7.2600109 (PMC12924001; doi:10.2807/1560-7917.ES.2026.31.7.2600109)
Supplement: Supplement [file 26-00109_KISSLING_Supplement.pdf]

Supplementary material

This supplementary material is hosted by *Eurosurveillance* as supporting information alongside the article “'Influenza vaccine effectiveness from nine studies during drifted A(H3N2) subclade K predominance in Europe, September 2025 to January 2026”, on behalf of the authors, who remain responsible for the accuracy and appropriateness of the content. The same standards for ethics, copyright, attributions and permissions as for the article apply. Supplements are not edited by *Eurosurveillance* and the journal is not responsible for the maintenance of any links or email addresses provided therein.

Supplementary Table S1. Study population characteristics of influenza A, influenza A(H3N2) and influenza A(H1N1)pdm09 cases, and test-negative controls, Denmark primary care study, 13 Oct 2025–16 Jan 2026

Characteristics	Test-negative controls (N = 19019)[a] n (%)	Influenza A cases (N = 4634) n (%)	Influenza A(H1N1)pdm09 cases (N = 293) n (%)	Influenza A(H3N2) cases (N = 813) n (%)
**Age (years)**				
Median (IQR)	50 (26-68)	31 (12-56)	48 (20-61)	26 (11-47)
**Age group (years)**				
0-4	1950 (10)	595 (13)	28 (10)	99 (12)
5-17	1428 (8)	1000 (22)	37 (13)	215 (26)
18-64	10053 (53)	2308 (50)	173 (59)	411 (51)
65+	5588 (29)	731 (16)	55 (19)	88 (11)
**Sex**				
Female	10767 (57)	2570 (55)	168 (57)	472 (58)
Male	8252 (43)	2064 (45)	125 (43)	341 (42)
**Underlying medical condition**				
Absence of underlying medical condition	11464 (60)	3285 (71)	204 (70)	610 (75)
Presence of underlying medical condition	7555 (40)	1349 (29)	89 (30)	203 (25)
**2025/26 seasonal influenza vaccination**				
No	13807 (73)	3865 (83)	246 (84)	716 (88)
Yes	5212 (27)	769 (17)	47 (16)	97 (12)

Abbreviation: IQR, interquartile range.

[a]Controls for 'influenza A' used here (number of controls differs slightly for influenza (sub)type-specific-analyses, due to the inclusion criteria).

Supplementary Table S2. Study population characteristics of influenza A, influenza A(H3N2) and influenza A(H1N1)pdm09 cases, and test-negative controls, I-MOVE primary care study, Europe, 29 Sep 2025–13 Jan 2026

Characteristics	Test-negative controls (N = 9690)[a] n (%)	Influenza A cases (N = 4930) n (%)	Influenza A(H1N1)pdm09 cases (N = 967) n (%)	Influenza A(H3N2) cases (N = 3317) n (%)
**Age (years)**				
Median (IQR)	34 (9-55)	22 (8-47)	39 (10-55)	19 (8-43)
**Age group (years)**				
0–4	1754 (18)	672 (14)	131 (14)	467 (14)
5–17	1530 (16)	1563 (32)	206 (21)	1132 (34)
18–64	5012 (52)	2256 (46)	509 (53)	1479 (45)
65+	1394 (14)	437 (9)	121 (13)	239 (7)

Characteristics	Test-negative controls (N = 9690)[a] n (%)	Influenza A cases (N = 4930) n (%)	Influenza A(H1N1)pdm09 cases (N = 967) n (%)	Influenza A(H3N2) cases (N = 3317) n (%)
**Sex**				
Female	5373 (55)	2518 (51)	488 (51)	1693 (51)
Male	4317 (45)	2410 (49)	479 (50)	1624 (49)
**Underlying medical condition**				
Absence of underlying medical condition	7035 (73)	3884 (79)	735 (76)	2645 (80)
Presence of underlying medical condition	2655 (27)	1044 (21)	232 (24)	672 (20)
**2025/26 seasonal influenza vaccination**				
No	7927 (82)	4265 (87)	830 (86)	2888 (87)
Yes	1763 (18)	663 (14)	137 (14)	429 (13)

Abbreviation: IQR, interquartile range.

[a]Controls for 'influenza A' used here (number of controls differs slightly for influenza (sub)type-specific-analyses, due to the inclusion criteria).

Supplementary Table S3. Study population characteristics of influenza A, influenza A(H3N2) and influenza A(H1N1)pdm09 cases, and test-negative controls, United Kingdom primary care study, 29 Sep 2025–04 Jan 2026

Characteristics	Test-negative controls (N = 10764)[a] n (%)	Influenza A cases (N = 4012) n (%)	Influenza A(H1N1)pdm09 cases (N = 273) n (%)	Influenza A(H3N2) cases (N = 3488) n (%)
**Age (years)**				
Median (IQR)	43 (23–63)	30 (15–53)	29 (14–51)	46 (28–61)
**Age group (years)**				
0–4	703 (7)	224 (6)	16 (6)	195 (6)
5–17	1338 (12)	1050 (26)	28 (10)	963 (28)
18–64	6326 (59)	2194 (55)	181 (66)	1882 (54)
65+	2397 (22)	544 (14)	48 (18)	448 (13)
**Sex**				
Female	6783 (63)	2399 (60)	174 (64)	2074 (59)
Male	3981 (37)	1613 (40)	99 (36)	1414 (41)
**Underlying medical condition**				
Absence of underlying medical condition	4697 (44)	1717 (43)	105 (38)	1534 (44)
Presence of underlying medical condition	2663 (25)	649 (16)	65 (24)	567 (16)
unknown	3404 (32)	1646 (41)	103 (38)	1387 (40)
**2025/26 seasonal influenza vaccination**				

Characteristics	Test-negative controls (N = 10764)[a] n (%)	Influenza A cases (N = 4012) n (%)	Influenza A(H1N1)pdm09 cases (N = 273) n (%)	Influenza A(H3N2) cases (N = 3488) n (%)
No	7711 (72)	3153 (79)	217 (79)	2748 (79)
Yes	3053 (28)	859 (21)	56 (21)	740 (21)

Abbreviation: IQR, interquartile range.

[a]Controls for 'influenza A' used here (number of controls differs slightly for influenza (sub)type-specific-analyses, due to the inclusion criteria).

Supplementary Table S4. Study population characteristics of influenza A, influenza A(H3N2) and influenza A(H1N1)pdm09 cases, and test-negative controls, Denmark hospital study, 13 Oct 2025–16 Jan 2026

Characteristics	Test-negative controls (N = 17469)[a] n (%)	Influenza A cases (N = 1515) n (%)	Influenza A(H1N1)pdm09 cases (N = 107) n (%)	Influenza A(H3N2) cases (N = 185) n (%)
Age (years)				
Median (IQR)	75 (61-83)	72 (53-81)	74 (64-82)	68 (44-81)
Age group (years)				
0-4	763 (4)	82 (5)	2 (2)	10 (5)
5-17	253 (1)	67 (4)	2 (2)	9 (5)
18-64	4160 (24)	435 (29)	23 (22)	61 (33)
65+	12293 (70)	931 (61)	80 (75)	105 (57)
Sex				
Female	8514 (49)	758 (50)	51 (48)	91 (49)
Male	8955 (51)	757 (50)	56 (52)	94 (51)
Underlying medical condition				
Absence of underlying medical condition	4347 (25)	500 (33)	32 (30)	76 (41)
Presence of underlying medical condition	13122 (75)	1015 (67)	75 (70)	109 (59)
2025/26 seasonal influenza vaccination				
No	10099 (58)	842 (56)	74 (69)	103 (56)
Yes	7370 (42)	673 (44)	33 (31)	82 (44)

Abbreviation: IQR, interquartile range.

[a]Controls for 'influenza A' used here (number of controls differs slightly for influenza (sub)type-specific-analyses, due to the inclusion criteria).

Supplementary Table S5. Study population characteristics of influenza A, influenza A(H3N2) and influenza A(H1N1)pdm09 cases, and test-negative controls, England emergency department study, 29 Sep 2025–04 Jan 2026

Characteristics	Test-negative controls (N = 105718)[a] n (%)	Influenza A cases (N = 32393) n (%)	Influenza A(H1N1)pdm09 cases (N = 330) n (%)	Influenza A(H3N2) cases (N = 3472) n (%)
**Age (years)**				
Median (IQR)	67 (41-80)	41 (15-73)	60 (28-77)	29 (10-67)
**Age group (years)**				
0-4	6350 (6)	3397 (10)	35 (11)	488 (14)
5-17	6232 (6)	5537 (17)	23 (7)	759 (22)
18-64	36991 (35)	12458 (38)	129 (39)	1275 (37)
65+	56145 (53)	11001 (34)	143 (43)	950 (27)
**Sex**				
Female	54781 (52)	17733 (55)	186 (56)	1893 (55)
Male	50937 (48)	14660 (45)	144 (44)	1579 (45)
**Underlying medical condition**				
Absence of underlying medical condition	37004 (35)	16799 (52)	132 (40)	1905 (55)
Presence of underlying medical condition	68714 (65)	15594 (48)	198 (60)	1567 (45)
**2025/26 seasonal influenza vaccination**				
No	63998 (61)	23746 (73)	239 (72)	2692 (78)
Yes	41720 (39)	8647 (27)	91 (28)	780 (22)

Abbreviation: IQR, interquartile range.

[a]Controls for 'influenza A' used here (number of controls differs slightly for influenza (sub)type-specific-analyses, due to the inclusion criteria).

Supplementary Table S6. Study population characteristics of influenza A, influenza A(H3N2) and influenza A(H1N1)pdm09 cases, and test-negative controls, England hospital study, 29 Sep 2025–14 Dec 2025

Characteristics	Test-negative controls (N = 24732)[a] n (%)	Influenza A cases (N = 9598) n (%)	Influenza A(H1N1)pdm09 cases (N = 184) n (%)	Influenza A(H3N2) cases (N = 1350) n (%)
**Age (years)**				
Median (IQR)	67 (41-80)	49 (17-74)	61 (43-76)	34 (12-68)
**Age group (years)**				
0-4	2090 (8)	925 (10)	15 (8)	171 (13)
5-17	1520 (6)	1496 (16)	6 (3)	274 (20)
18-64	7626 (31)	3601 (38)	85 (46)	524 (39)
65+	13496 (55)	3576 (37)	78 (42)	381 (28)
**Sex**				
Female	12519 (51)	5420 (56)	99 (54)	767 (57)
Male	12213 (49)	4178 (44)	85 (46)	583 (43)
**Underlying medical condition**				

Characteristics	Test-negative controls (N = 24732)[a] n (%)	Influenza A cases (N = 9598) n (%)	Influenza A(H1N1)pdm09 cases (N = 184) n (%)	Influenza A(H3N2) cases (N = 1350) n (%)
Absence of underlying medical condition	8019 (32)	4286 (45)	56 (30)	644 (48)
Presence of underlying medical condition	16713 (68)	5312 (55)	128 (70)	706 (52)
**2025/26 seasonal influenza vaccination**				
No	17660 (71)	7429 (77)	150 (82)	1094 (81)
Yes	7072 (29)	2169 (23)	34 (18)	256 (19)

Abbreviation: IQR, interquartile range.

[a]Controls for 'influenza A' used here (number of controls differs slightly for influenza (sub)type-specific-analyses, due to the inclusion criteria).

Supplementary Table S7. Study population characteristics of influenza A, influenza A(H3N2) and influenza A(H1N1)pdm09 cases, and test-negative controls, I-MOVE hospital study, Europe, 16 Sep 2025 –11 Jan 2026

Characteristics	Test-negative controls (N = 4997)[a] n (%)	Influenza A cases (N = 2142) n (%)	Influenza A(H1N1)pdm09 cases (N = 369) n (%)	Influenza A(H3N2) cases (N = 986) n (%)
**Age (years)**				
Median (IQR)	69 (20–81)	73 (55–82)	74 (63–82)	71 (42–81)
**Age group (years)**				
0–4	1023 (20)	178 (8)	21 (6)	99 (10)
5–17	212 (4)	123 (6)	8 (2)	76 (8)
18–64	968 (19)	425 (20)	74 (20)	211 (21)
65+	2794 (56)	1416 (66)	266 (72)	600 (61)
**Sex**				
Female	2375 (48)	1046 (49)	180 (49)	512 (52)
Male	2622 (52)	1096 (51)	189 (51)	474 (48)
**Underlying medical condition**				
Absence of underlying medical condition	1853 (37)	695 (32)	96 (26)	365 (37)
Presence of underlying medical condition	4290 (86)	1447 (68)	273 (74)	621 (63)
**2025/26 seasonal influenza vaccination**				
No	3192 (64)	1380 (64)	246 (67)	637 (65)
Yes	1805 (36)	762 (36)	123 (33)	349 (35)

Abbreviation: IQR, interquartile range.

[a]Controls for 'influenza A' used here (number of controls differs slightly for influenza (sub)type-specific-analyses, due to the inclusion criteria).

Supplementary Table S8. Study population characteristics of influenza A, influenza A(H3N2) and influenza A(H1N1)pdm09 cases, and test-negative controls, Scotland hospital study, 28 Sep 2025–21 Jan 2026

Characteristics	Test-negative controls (N = 27635)[a] n (%)	Influenza A cases (N = 3619) n (%)	Influenza A(H1N1)pdm09 cases (N = 58) n (%)	Influenza A(H3N2) cases (N = 703) n (%)
Age (years)				
Median (IQR)	70 (53, 80)	67 (38, 78)	53 (6, 77)	19 (6, 71)
Age group (years)				
0-4	1134 (4.1%)	230 (6.4%)	11 (19%)	127 (18%)
5-17	1270 (4.6%)	300 (8.3%)	9 (16%)	220 (31%)
18-64	8793 (32%)	1153 (32%)	13 (22%)	148 (21%)
65+	16438 (59%)	1936 (53%)	25 (43%)	208 (30%)
Sex				
Female	14602 (53%)	2005 (55%)	35 (60%)	374 (53%)
Male	13033 (47%)	1614 (45%)	23 (40%)	329 (47%)
**Underlying medical condition**				
Absence of underlying medical condition	6124 (22%)	1006 (28%)	24 (41%)	349 (50%)
Presence of underlying medical condition	21511 (78%)	2613 (72%)	34 (59%)	354 (50%)
**2025/26 seasonal influenza vaccination**				
No	19859 (72%)	2652 (73%)	47 (81%)	561 (80%)
Yes	7776 (28%)	967 (27%)	11 (19%)	142 (20%)

Abbreviation: IQR, interquartile range.

[a]Controls for 'influenza A' used here (number of controls differs slightly for influenza (sub)type-specific-analyses, due to the inclusion criteria).

Supplementary Table S9. Study population characteristics of influenza A, influenza A(H3N2) and influenza A(H1N1)pdm09 cases, and test-negative controls, Northern Ireland hospital study, 29 Sep 2025–10 Jan 2026

**Characteristics**	**Test-negative controls (N = 8597)[a] n (%)**	**Influenza A cases (N = 1374) n (%)**	**Influenza A(H1N1)pdm09 cases (N = 40) n (%)**	**Influenza A(H3N2) cases (N = 381) n (%)**
**Age (years)**				
Median (IQR)	73 (60-82)	74 (62-82)	75 (65-81)	73 (61-82)
**Age group (years)**				
0-4	0 (0)	0 (0)	0 (0)	0 (0)
5-17	0 (0)	0 (0)	0 (0)	0 (0)
18-64	2794 (32)	421 (31)	10 (25)	127 (33)
65+	5803 (68)	953 (69)	30 (75)	254 (67)

Characteristics	Test-negative controls (N = 8597)[a] n (%)	Influenza A cases (N = 1374) n (%)	Influenza A(H1N1)pdm09 cases (N = 40) n (%)	Influenza A(H3N2) cases (N = 381) n (%)
**Sex**				
Female	4487 (52)	756 (55)	21 (52)	210 (55)
Male	4110 (48)	618 (45)	19 (48)	171 (45)
**Underlying medical condition**				
Absence of underlying medical condition	NA		NA	NA
Presence of underlying medical condition	NA		NA	NA
**2025/26 seasonal influenza vaccination**				
No	5940 (69)	856 (62)	24 (60)	241 (63)
Yes	2657 (31)	518 (38)	16 (40)	140 (37)

Abbreviation: IQR, interquartile range.

[a]Controls for 'influenza A' used here (number of controls differs slightly for influenza (sub)type-specific-analyses, due to the inclusion criteria).

Supplementary Figure S1. Interim vaccine effectiveness estimates against any influenza, by age group and target population, seven European studies, interim influenza season 2025/26

(a) All influenza

Study	Population[a]	Cases N	Cases vacc	Cases %	Controls N	Controls vacc	Controls %	VE (95% CI)[b]
Primary care								
EU-PC	All ages	4938	663	13	9690	1763	18	37 (30–44)
	0–17 years [c]	2241	255	11	3284	582	18	47 (37–56)
	18–64 years	2260	198	9	5012	490	10	26 (10–39)
	65 years and above	437	210	48	1394	691	50	40 (23–53)
	Target group [d]	2160	551	26	5067	1553	31	37 (28–45)
UK-PC	All ages (≥ 2 years)	4023	862	21	10764	3053	28	40 (33–46)
	2–17 years	1279	232	18	2041	662	32	59 (51–66)
	18–64 years	2200	289	13	6326	1082	17	32 (20–43)
	65 years and above	544	341	63	2397	1309	55	25 (6–40)
Hospital								
EN-ED[e]	All ages (≥ 2 years)	32524	8675	27	105718	41720	39	38 (36–40)
	2–17 years	8969	1120	12	12582	4045	32	70 (68–72)
	18–64 years	12526	1812	14	36991	7817	21	29 (24–33)
	65 years and over	11029	5743	52	56145	29858	53	29 (25–32)
EN-H [e]	All ages (≥ 2 years)	9640	2182	23	24732	7072	29	42 (38–45)
	2–17 years	2432	244	10	3610	913	25	71 (66–76)
	18–64 years	3619	523	14	7626	1195	16	28 (18–36)
	65 years and above	3589	1415	39	13496	4964	37	36 (30–41)
EU-H	All ages	2213	792	36	5004	1807	36	33 (25–41)
	0–17 years	307	24	8	1236	240	19	69 (51–82)
	18–64 years	441	74	17	969	175	18	22 (-8–43)
	65 years and above	1465	694	47	2799	1392	50	27 (16–37)
	Target group [d]	2011	784	39	4297	1772	41	33 (25–41)
NI-H	All adults (≥ 18 years)	1374	518	38	8597	2657	31	21 (11–31)
	18–64 years	421	84	20	2794	418	15	4 (-26–27)
	65 years and above	953	434	46	5803	2239	39	27 (16–37)
SC-H	All ages (≥ 2 years)	4397	1125	26	27635	7776	28	34 (27–39)
	2–17 years	904	89	10	2404	489	20	61 (41–74)
	18–64 years	1321	146	11	8793	988	11	23 (6–38)
	65 years and over	2172	890	41	16438	6299	38	35 (28–41)

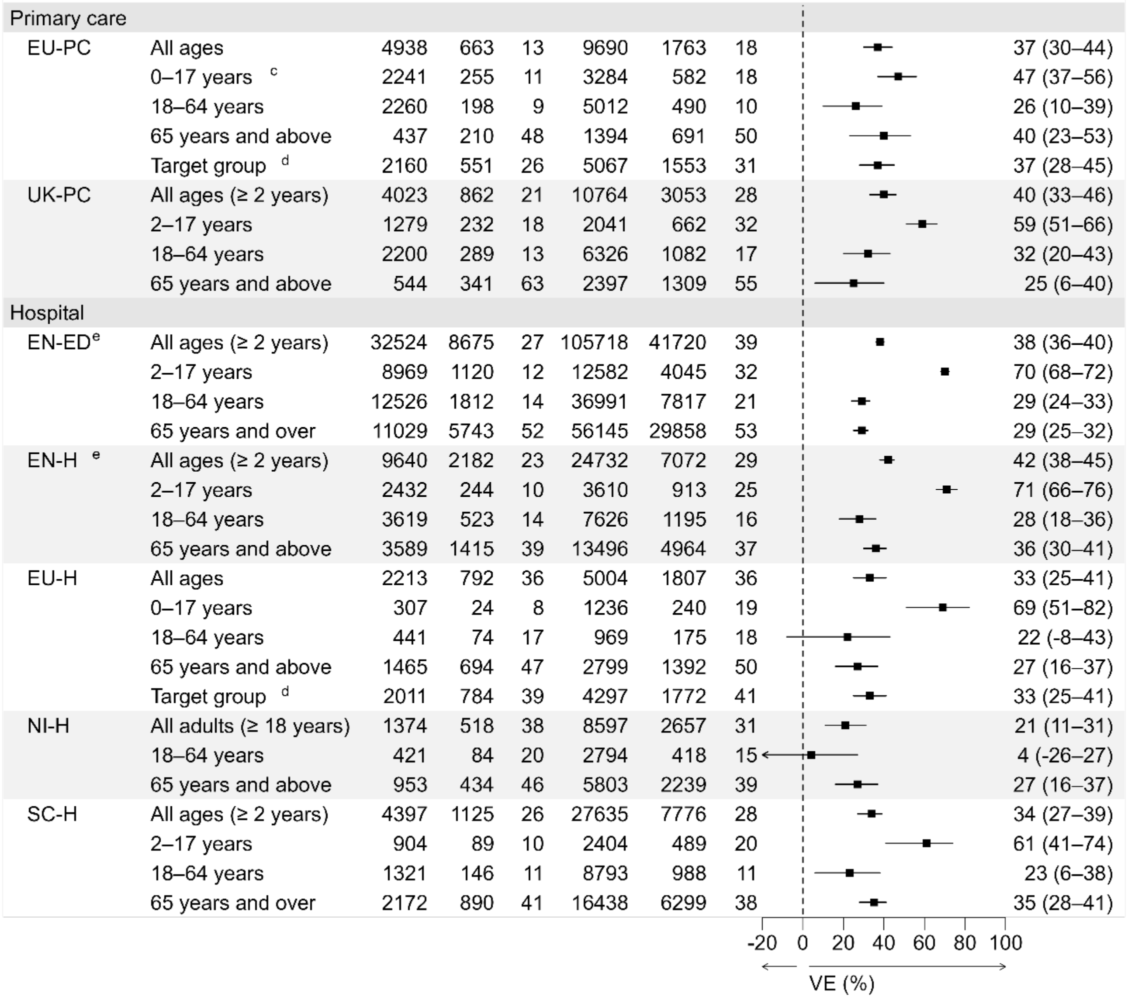

CI: confidence interval; EN-ED: England emergency department study; EN-H: England hospital study; EU: European Union; EU-H/EU-PC: EU hospital/primary care multicentre I-MOVE studies; I-MOVE: Influenza – Monitoring of Vaccine Effectiveness; N: number; PC: primary care; NI-H: Northern Ireland hospital study; SC-H: Scottish hospital study; UK-PC: United Kingdom multicentre primary care study; vacc: vaccinated; VE: vaccine effectiveness.

[a] Age- or target group–specific VE estimates were not available for some study sites owing to insufficient sample size. Estimates not provided for DK-H and DK-PC.

[b] Adjustment variables are described in Table 1.

[c] In EU-PC, the age group 0–17 years includes children aged ≥ 6 months to 17 years.

[d] Target groups for seasonal influenza vaccination were defined locally by each study site.

[e] There is a 50–73% overlap among EN-ED and EN-H patients.

Supplementary Figure S2. Interim vaccine effectiveness estimates against influenza B, by age group and target population, two European studies, interim influenza season 2025/26

(b) Influenza B

Study	Population[a]	Cases N	Cases vacc	Cases %	Controls N	Controls vacc	Controls %	VE (95% CI)[b]
Hospital								
EN-ED[c]	All ages (≥ 2 years)	180	44	24	105718	41720	39	35 (6–56)
	2–17 years	46	11	NC	12582	4045	32	35 (-29–70)
	18–64 years	89	7	8	36991	7817	21	62 (21–84)
	65 years and over	45	26	NC	56145	29858	53	15 (-64–55)
EN-H[c]	All ages (≥ 2 years)	55	17	NC	24732	7072	28.6	-10 (-106–43)

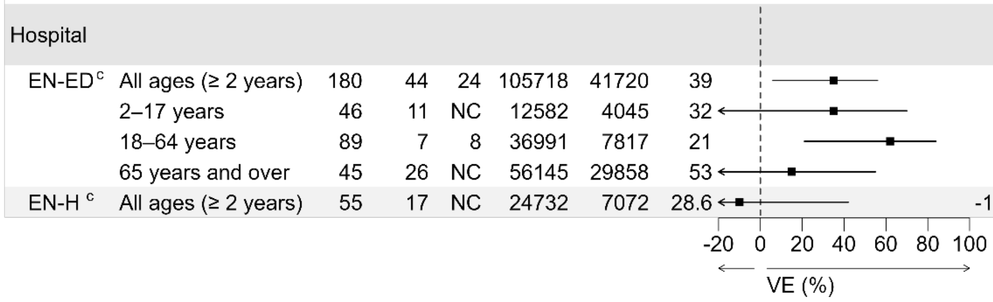

CI: confidence interval; EN-ED: England emergency department study; EN-H: England hospital study; EU: European Union; VE: vaccine effectiveness.

[a] Age-specific VE estimates were not available for some study sites owing to insufficient sample size.

[b] Adjustment variables are described in Table 1 in the main text.

[c] There is a 50–73% overlap among EN-ED and EN-H patients.
